# Somatotype Profiles of Montenegrin Karatekas: An Observational Study

**DOI:** 10.3390/ijerph182412914

**Published:** 2021-12-07

**Authors:** Jelena Slankamenac, Dusko Bjelica, Damjan Jaksic, Tatjana Trivic, Miodrag Drapsin, Sandra Vujkov, Toni Modric, Zoran Milosevic, Patrik Drid

**Affiliations:** 1 Faculty of Sport and Physical Education, University of Novi Sad, 21000 Novi Sad, Serbia; jelly.95@live.com (J.S.); jaksic_damjan@yahoo.com (D.J.); ttrivic@yahoo.com (T.T.); zoranaisns29@gmail.com (Z.M.); 2 Faculty of Sports and Physical Education, University of Montenegro, 81400 Nikšić, Montenegro; montenegrosportmont@t-com.me; 3 Faculty of Medicine, University of Novi Sad, 21000 Novi Sad, Serbia; miodrag.drapsin@mf.uns.ac.rs; 4 College of Vocational Studies for the Education of Preschool Teachers and Sports Trainers, 24000 Subotica, Serbia; sandravujkov@vsovsu.rs; 5 Faculty of Kinesiology, University of Split, 21000 Split, Croatia; toni.modric@kifst.hr

**Keywords:** karate, kumite, weight categories, anthropometry, body composition, martial arts, combat sports

## Abstract

Competitive karate activity involves numerous factors affecting performance in sport. Physical structure and somatotype is considered to be one of them. This study aimed to determine whether there are differences between karate athletes in five male and five female official weight categories in different anthropometric measurements and to determine the somatotype profiles of athletes divided by weight categories. This study consisted of a total of 27 male karate athletes (21.88 ± 4.66 years) and 24 female karate athletes (20.29 ± 3.14 years). Measurements were taken in April 2020. Athletes are classified into official weight categories according to World Karate Federation rules. Somatotypes were calculated using anthropometry. One-way analysis of variance and Tukey’s post hoc tests were used for statistical analysis to compare group differences regarding weight categories. Anthropometric parameters were highest in the heaviest categories compared to lighter categories. All male subjects were endomorphic mesomorph, except for category <84 kg, which was endomorphic ectomorphs. Somatotype analysis of male categories found a difference between the <75 kg and <84 kg in endomorphy. In mesomorphy, there is no difference between categories. Perceiving ectomorphy, there is a significant difference between the first category and the >84 kg. Profiling female athletes, three different types of somatotypes were obtained concerning the weight category. The lightest weight category was predominantly endomorphic ectomorphs, and two weight categories were ectomorphic endomorphs (<61 kg and <68 kg), and the other two weight categories were endomorphic mesomorphs (<55 kg and >68 kg). Somatotype differences in the female karate athletes were observed only in the ectomorphy components, between <50 kg and <61 kg. The present study points to how the somatotypes profiles of karate athletes differ between weight categories.

## 1. Introduction

The origin of karate remains hidden by opaque veil legends, but we still know that karate originates from the Far East, and it was widely practiced by the people who were followers of such different religions as Buddhism, Islam, Hinduism, and Taoism. It was first developed in Okinawa, Japan, in the 17th century when the Japanese took this island and prohibited the usage of all weapons [1]. It gained popularity after the Second World War. Karate is one of the most popular and widely practiced martial arts of today, and only in 2021 (Tokyo, Japan) had its appearance in the Olympic games. It is characterized by two distinguished competitive disciplines: *Kata* and *Kumite* (sports fight). *Kata* means form, and it is a predetermined series of offensive and defensive techniques and movements in standard order, versus one or more nonexistent opponents. Fundamental elements of the *Kata* technique involve rhythm, expressiveness, and *Kime* (a short isometric muscle contraction performed when a technique is finished) [2]. Karatekas that outreach the final are obligated to perform one *Tokui* (free-style *Kata*) and one *Shitei* (fixed *Kata* styles). Athletes have 60–80 s to complete the *Kata* [3]. *Kumite*, on the other hand, represents combat between two karate athletes under certain rules. Strikes are limited to determining areas: face, head, neck, chest, abdomen, side, and back. The duration of the *Kumite* match is 3 min for male and female senior athletes [3]. Judges score kicks and punches—*Ippon* (3 points), *Waza-ari* (2 points), and *Yuko* (1 point). Points are awarded when a technique is executed according to the following principle: good form, vigorous application, sporting attitude, awareness, correct distance, and good timing. *Kumite* competitors are divided into five weight categories for both males and females (<60 kg, <67 kg, <75 kg, <84 kg and >84 kg for males and <50 kg, <55 kg, <61 kg, <68 kg and >68 kg for females). Weight categories in karate and other combat sports can ensure fair competition by complementary opponents of similar body mass and stature [4].

One of the oldest questions in every sport is “what actually makes a successful athlete successful?” Morphological features play an important role in accomplishments in most sports. Body form provides a foundation for the improvement of movement technique and particular physical fitness. When selecting athletes in a particular sport, it is observed whether their physical characteristics fit with a “model” somatic pattern for that sport. That model is based on somatic patterns recorded in athletes who have systematically achieved the best results. Assessment of body composition consists of an assessment of the somatotype, which is based on the relationship between body fat and the lean body content, muscular development, skeleton robustness, and reciprocal ponderal index (height divided by the cube root of body weight) [5,6,7,8]. The most commonly used technique in somatotype assessment is the Heath and Carter method [9]. They emphasize that the somatotype is defined as representing the individual’s present morphological conformation. Heath-Carter method is used primarily in its anthropometric form in practice, and it is best suited for sports science. Anthropometric measurements are objective and can show body shape, composition, and proportionality. The somatotype consists of three main components in relation to body height: endomorphy, mesomorphy, and ectomorphy [10]. Endomorphy is the first component, and it represents relative fatness or leanness. The second component is mesomorphy and this shows relative musculoskeletal development adjusted for height. Ectomorphy, the third component, is the relative linearity of the build [5]. The knowledge of these characteristics is most informative for coaches and athletes.

Very often, the physical structure is considered as one of the elements for high performance in many sports, as well as in competitive karate [11,12]. In karate, empirical experience states that the athlete’s body height and longitudinal dimensions, such as arm and leg length, are some of the main advantages of karate athletes because these measures allow karatekas to raise their legs higher during the kick and they can fight from greater distances [13]. Comparing karate athletes with the general population, they are distinguished by muscular mass with enhanced transverse skeleton dimensionality and reduced adipose tissue. It is known that the body composition of athletes has a great impact on achieving top sports results. Up to date, several studies have dealt with somatotypes in male karate athletes [14,15,16]. However, there is a lack of evidence regarding female karate somatotype. With this in mind, anthropometric parameters were measured, and the somatotypes of both male and female Montenegrin karatekas were determined.

This study aimed to determine whether there are differences between karate athletes in five male and five female weight categories in different anthropometric measurements and to determine the somatotype profiles of athletes. The results of this study should provide a more specific outline of the morphological biotype best suited to the specific technical requirements for *Kumite* athletes of both genders.

## 2. Materials and Methods

### 2.1. Subjects

A total of 60 senior karate athletes from Montenegro participated in the National Championships in 2020. For the purpose of this study, we have chosen 51 karate athletes (black belt). According to the calculation, considering that the five weight categories are analyzed, the total sample size should be much larger. However, in this specific case, the total population is 60 competitors, so the classical formula cannot be applied. A cohort of 27 male (21.9 ± 4.7years) and 24 female karate athletes (20.3 ± 3.14 years) of a national level volunteered in this cross-sectional study. The subject sample included healthy, black belt karate senior athletes, with no prior injuries, minimum five year training experience and overall weekly training volume of over 20 h. Measurements were taken in April 2020. All testing procedures were conducted during the karate camp ahead of the National Championship held in Nikšić (Montenegro). Participants were divided into five official male categories <60 kg (*n* = 5), <67 kg (*n* = 8), <75 kg (*n* = 6), <84 kg (*n* = 4), and >84 kg (*n* = 4) and five female weight categories <50 kg (*n* = 2), <55 kg (*n* = 7), <61 kg (*n* = 7), <68 kg (*n* = 6), and >68 kg (*n* = 2) in accordance with their current body mass, age and gender [3]. All athletes were introduced to all of the testing procedures applied in the current research. All anthropometrical measurements were taken from the participants in the same position, in the morning hours (before breakfast), by the same two experienced graduated students of the Faculty for Sport and Physical Education, University of Montenegro. Informed written consent was acquired from each subject, and all procedures were executed and conducted according to the guidelines of the Declaration of Helsinki and approved by the Institutional Review Board of the Faculty of Sport and Physical Education University of Novi Sad, Serbia (Ref. No. 46-06-02/2020-1).

### 2.2. Anthropometrical Measurements

In order to determine somatotypes, ten required measurements were taken as follows: body height and body mass, bi-epicondylar breadths of humerus and femur, four skinfold measurements (triceps, supraspinal, subscapular, and medial calf), and two girths (arm and calf). Body height (cm) was determined using a Martin anthropometer (GPM, Bachenbülach, Switzerland); body mass (kg) was measured with an electronic scale (SECA, Hamburg, Germany) with a sensitivity level of 0.1 kg; skinfolds were taken on the right side of the body using a John Bull caliper (British Indicator Ltd., Weybridge, UK), accurate to 0.2 mm; circumference measurements (cm) were obtained with a steel measuring tape, and wrist girth and bi-epicondylar diameters of the femur and humerus (mm) were measured using a small spreading caliper (SiberHegner, Zurich, Switzerland). Somatotypes were determined using the Carter and Heath method [9].

### 2.3. Statistical Analysis

The data obtained are presented as standard deviation (±) and means. One-way analysis of variance (ANOVA) and Tukey’s post hoc tests was used to compare group the differences by weight categories. Furthermore, the effect size (h2) was calculated. The level of significance was set at *p*-value < 0.05. SPSS statistics software was used to conduct analyses.

## 3. Results

The study involved 27 male and 24 female Montenegrin karate athletes. Anthropometric characteristics and somatotype parameters were measured and presented in tables and charts. Both males and females were divided into five weight categories (male: <60 kg, <67 kg, <75 kg, <84 kg and >84 kg; female: <50 kg, <55 kg, <61 kg, <68 kg and >68 kg). Anthropometric parameters increased within the weight category. 

Statistically significant differences in male categories were found between the first category (<60 kg) in body height compared to the last three categories (<75 kg, <84 kg, and >84 kg). The highest athletes were in the <84 kg category. There was no significant difference found between groups in breadths of humerus and femur. In term of arm girths, there were differences between <60 kg, <67 kg and >84 kg. However, a difference between <60 kg and the last three categories (<75 kg, <84 kg, and >84 kg) in terms of calf circumference was found. 

Measuring skinfolds, statistically significant differences were shown only between <84 kg and the first three groups (<60 kg, <67 kg, and <75 kg) in supraspinal skinfold. Other differences in skinfolds were not at a significant level (Table 1).

**Figure 1:**
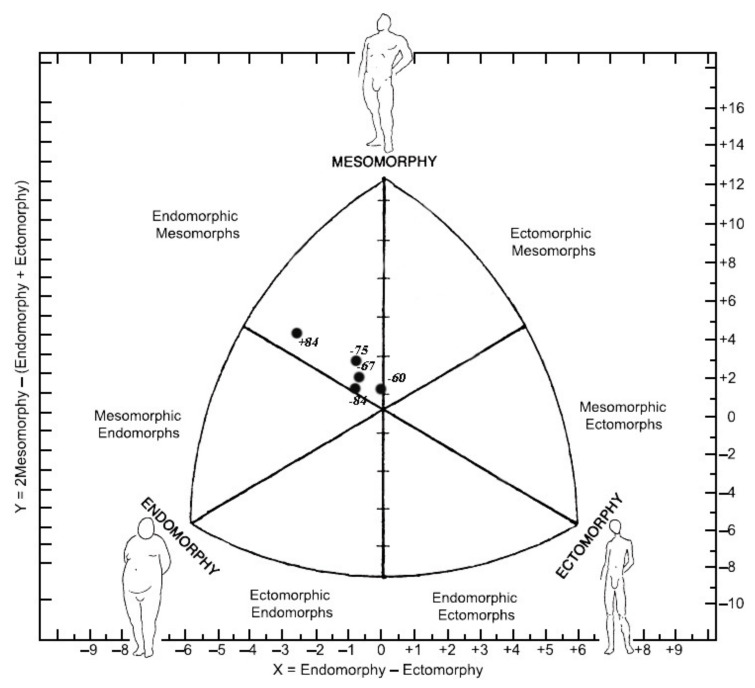
Somatochart of male karate athletes by weight categories.

Somatotype analysis of male categories found a difference between the <75 kg and <84 kg in endomorphy. In mesomorphy, there is no difference between the categories. Perceiving ectomorphy, there is a significant difference between the first category and the >84 kg. All male subjects were endomorphic mesomorph, except for category <84 kg, which was endomorphic ectomorphs (Figure 1).

In the female groups, body height increased in relation to the weight category and differed significantly between <50 kg, <55 kg, <61 kg, and the heaviest group (>68 kg), and between the first two (<50 kg, and <55 kg) and <68 kg. The breadth of the humerus shows a difference between >68 kg and all of the other groups (<50 kg, <55 kg, <61 kg, <68 kg). The only difference in the breadth of the femur is between the lightest <50 kg) and the heaviest (>68 kg) category. Measuring arm circumference, there is one difference, between the <50 kg and >68 kg categories. Additionally, there is one difference between the groups in the circumference of the calf, between <50 kg and >68 kg. The categories did not differ significantly in terms of the thickness of the skin folds (Table 2).

**Figure 2:**
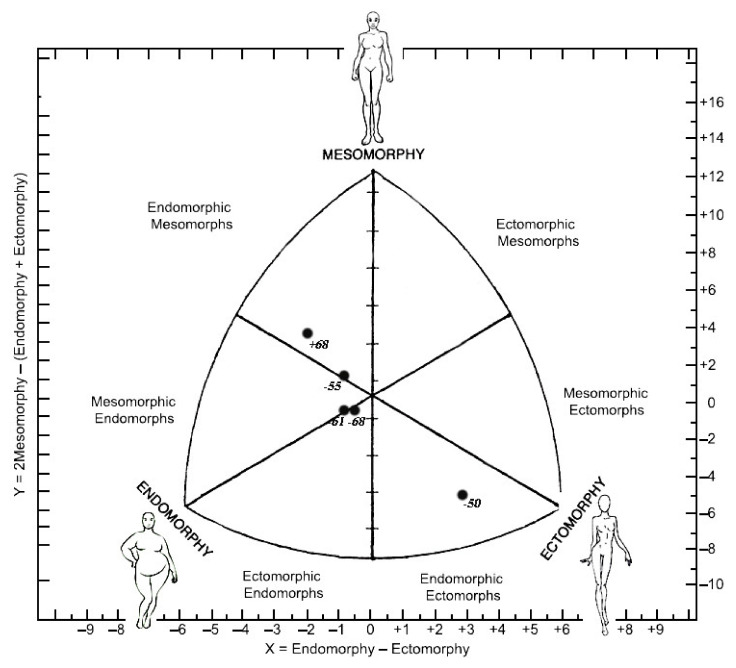
Somatochart of female karate athletes by weight categories.

The somatochart showed that the lightest weight category was predominantly endomorphic ectomorphs. Two weight categories were ectomorphic endomorphs (<61 kg and <68 kg), and the other two weight categories were endomorphic mesomorphs (<55 kg and >68 kg). Somatotype differences in the female karate athletes were observed in the ectomorphy components, between <50 kg and <61 kg, and in mesomorphy between <50 kg and >68 kg. (Figure 2).

## 4. Discussion

Accomplishment in most sports depends on the physical, physiological, psychological, and social characteristics of the athlete [17]. This study is focused on physical characteristics of karatekas, and determining whether there is a difference in these characteristics between Montenegrin karatekas in different weight categories.

The somatotype profiles of male and female Montenegrin karate athletes were evaluated in relation to different weight categories. Study results have indicated several differences in somatotype for the female group and some anthropometric characteristics throughout weight categories for both female and male groups of observed karate athletes. Somatotypes of male karatekas were mostly homogeneous. The obtained results showed the predominance of endomorphic mesomorphs, except for athletes in the <84 kg category, who were endomorphic ectomorphs. In contrast to our finding, some other recent studies found dominantly mesomorphic somatotypes in male karate athletes [18,19]. A higher mesomorphy component is significant in that increased muscle mass can be considered as an important benefit for athletes facing severe physical confrontation during training and competition, while increased fat mass reflected in endomorphism may prove useful in affecting absorption and dispersing such forces [20,21]. 

On the other hand, female karate athletes’ results showed different types of somatotypes. Profiling female athletes, three different types of somatotypes in relation to weight category were obtained. Female categories show that the lightest weight category was predominantly endomorphic ectomorphs. Two weight categories were ectomorphic endomorphs (<61 kg and <68 kg), and the other two weight categories were endomorphic mesomorphs (<55 kg and >68 kg). This finding is in accordance with other studies that also examined anthropometric characteristics. Fritzsche and Raschka [14] state that the karatekas who practice *Kata* are exhibit more endomorphs characteristics and *Kumite* athletes take more ectomorph positions in somatocharts. 

Karate athletes are characterized by a low percentage of fat tissue and a harmonic body constitution. However, different nationalities have different percentages of fat tissue [22]. A review of data from the literature discovered that elite karatekas are ectomorphic mesomorphs with a small amount of adipose tissue [23,24,25]. Prominent vertical skeletal development among top-level karatekas is the most influential anthropometric feature [23]. Controlling body composition is obligatory to clarify an athlete’s best weight category [26]. 

In the present study, statistically significant differences in male categories were found between the first two categories in body height compared to the last three categories. In the female groups, body height increased concerning the weight category and differed significantly between <50 kg, <55 kg, <61 kg, and the heaviest group, and in between the first two and <68 kg. Gloc et al. [26] obtained the results which proposed that taller karate athletes with a higher percentage of muscular mass had a better outcome. Morphological characteristics also influence specific motor skills in junior karate athletes [27]. Analysis of bone diameters showed no significant differences in male categories, and in female categories, there are differences in the humerus breadth between the heaviest and all of the other groups; femur breadth was different between the lightest and the heaviest weight category. Azary and Izadi [28] stated that elite karatekas have longer lower limbs compared to non-elite athletes, despite their similarity in body height. They imply that Iranian karatekas have a higher skelic index than Italian athletes. Throughout karate sparring, various techniques are executed, and all of them require explosiveness and high speed to perform. The athletes with longer longitudinal dimensions seem to possess a particular superiority for acquiring points before the opponent, and they are able to use longer limb length to get the upper hand facing the opponent in combat [29]. Skinfolds differed significantly between groups, neither in the male nor the female categories, except between <84 kg and the first three groups in the supraspinal skinfold in male categories. 

According to Przybylski et al. [30], the most significant qualificator factor for success in performing karate for each gender appears to be well-built strength based on the morphology of the limbs. In the current study, both male and female karatekas in the heaviest weight category differed significantly from the lighter categories in terms of anthropometric values.

One of the study limitations is presented by the relatively small number of athletes per each weight category. More athletes per each weight group could provide more detailed information regarding somatotypes in karate. Further investigation should be aimed on the dominant techniques typically used within categories and acquiring better insight into whether there is difference in the specific techniques that are used in relation to specific physical characteristics. Furthermore, it could be observed, additionally, whether specific techniques applied occur throughout various weight categories.

National level *Kumite* athletes of both genders in all weight categories were categorized by their physical characteristics in somatotypes in this study. Practicing karate seems to produce general morphological adaptation to the training process. Further studies are needed in order to investigate potential long-term adaptation in terms of the experience of athletes (i.e., national vs. international karatekas), as well as differences in somatotypes between *Kata* and *Kumite* athletes for both genders.

## 5. Conclusions

The findings of the study regarding somatotypes and anthropometric characteristics throughout various weight categories in karate should provide important information regarding future training processes, testing, as well as for the identification and selection of karate athletes. There are very few differences between karatekas in different weight categories. Differences were found between the heaviest and lighter categories in terms of body height, breadths, and girths in both male and female categories. There were no differences in the thickness of skin folds. Female categories show heterogeneous somatotypes, but the only significant difference was in ectomorphy between <50 kg and <61 kg. Male groups have similar somatotypes. Most of them were endomorphic mesomorphs. Significant differences between males were found in endomorphy (<67 kg and <84 kg) and in ectomorphy (<60 kg and >85 kg). By studying these characteristics, scientists can give specific details on the functional and morphological somatotype best suited for any sport. The present study could be significant for profiling and selecting karate athletes based on gender, age, and weight categories.

## Figures and Tables

**Table 1:** Differences between weight categories of male karatekas.

Male	−60 ^a^ (*n* = 5)	−67 ^b^ (*n* = 8)	−75 ^c^ (*n* = 6)	−84 ^d^ (*n* = 4)	+84 ^e^ (*n* = 4)	Statistics
Variable	M ± SD	M ± SD	M ± SD	M ± SD	M ± SD	
Body height (cm)	168 ± 8.2	176.6 ± 7.2	183.9 ± 2.7 ^a^	185.1 ± 3.5 ^a^	184.8 ± 6.4 ^a^	F = 7.16, ***p* = 0.001**, η^2^ = 0.57
	Breadths					
Humerus (cm)	7.3 ± 1	7.1 ± 0.6	7.6 ± 0.9	7.3 ± 0.2	7.4 ± 0.5	F = 0.31, *p* = 0.869, η^2^ = 0.05
Femur (cm)	9.2 ± 1	9.6 ± 0.4	10.2 ± 1	10.3 ± 0.5	10.1 ± 0.3	F = 1.98, *p* = 0.133, η^2^ = 0.26
	Girths					
Arm (cm)	23.5 ± 2.8	25.9 ± 1.1	27.4 ± 1.2	26.4 ± 1.5	29.2 ± 1.8 ^a,b^	F = 7.32, ***p* = 0.001**, η^2^ = 0.57
Calf (cm)	34.2 ± 3	36.6 ± 1.4	38.1 ± 2 ^a^	39.1 ± 1.7 ^a^	40.5 ± 2 ^a,b^	F = 6.59, ***p* = 0.001**, η^2^ = 0.55
	Skinfolds					
Triceps (mm)	9.4 ± 3.9	8.2 ± 2.4	6.3 ± 1.3	9.2 ± 1.2	8.4 ± 5.3	F = 0.90, *p* = 0.479, η^2^ = 0.14
Supraspinale (mm)	6.4 ± 1.8	7.5 ± 2.2	6.6 ± 2.2	14.9 ± 3.6 ^a,b,c^	11.1 ± 4.8	F = 7.33, ***p* = 0.001**, η^2^ = 0.57
Subscapular (mm)	7.4 ± 1.1	8.8 ± 1.3	8.3 ± 1	8.6 ± 1.1	10.8 ± 2.4	F = 3.53, *p* = 0.230, η^2^ = 0.39
Calf (mm)	9.5 ± 4.5	8.5 ± 2.6	6.7 ± 1.8	11 ± 2.6	9.5 ± 3.9	F = 1.32, *p* = 0.293, η^2^ = 0.19
	Somatotypes					
Endomorphy	2.3 ± 0.6	2.3 ± 0.6	1.9 ± 0.4	3.4 ± 1 ^c^	2.8 ± 1.2	F = 2.21, *p* = 0.100, η^2^ = 0.29
Mesomorphy	3.8 ± 1.5	3.7 ± 1.1	4 ± 1.2	2.5 ± 1.6	4.4 ± 1	F = 0.32, *p* = 0.857, η^2^ = 0.06
Ectomorphy	3.9 ± 1.3	3.1 ± 1.1	3.2 ± 0.3	2.6 ± 0.7	1.8 ± 1.2 ^a^	F = 2.81, *p* = 0.051, η^2^ = 0.34

Legend: M—Mean; SD—standard deviation; different from: ^a^—<60; ^b^—<67; ^c^—<75; ^d^—<84; ^e^—>85; significant differences in bold.

**Table 2:** Differences between weight categories of female karatekas.

Female	−50 ^a^ (*n* = 2)	−55 ^b^ (*n* = 7)	−61 ^c^ (*n* = 7)	−68 ^d^ (*n* = 6)	+68 ^e^ (*n* = 2)	Statistics
Variable	M ± SD	M ± SD	M ± SD	M ± SD	M ± SD	
Body height (cm)	161.5 ± 6.4	162.8 ± 4.7	163.8 ± 2.9	171.5 ± 5.5 ^b,c^	181.0 ± 0 ^a,b,c^	F = 9.66, ***p* = 0.000**, η^2^ = 0.67
	Breadths					
Humerus (cm)	5.6 ± 0.6	6.1 ± 0.2	6 ± 0.2	6.3 ± 0.9	7.8 ± 0.4 ^a,b,c,d^	F = 6.23, ***p* = 0.002**, η^2^ = 0.57
Femur (cm)	7 ± 2.8	8.9 ± 0.2	8.3 ± 0.9	9.2 ± 0.9	10.5 ± 0.7 ^a^	F = 3.99, ***p* = 0.016**, η^2^ = 0.46
	Girths					
Arm (cm)	21.6 ± 2.8	23.5 ± 1.1	23.5 ± 0.9	23.8 ± 0.6	26.7 ± 3.7 ^a^	F = 3.86, ***p* = 0.018**, η^2^ = 0.45
Calf (cm)	32.4 ± 0.1	35.1 ± 1.6	35.9 ± 1.4	36.8 ± 1.4	37.9 ± 3 ^a^	F = 4.49, ***p* = 0.010**, η^2^ = 0.49
	Skinfolds					
Triceps (mm)	10.4 ± 2.5	11.9 ± 2.2	14.1 ± 3.9	11.7 ± 1.1	10 ± 2.6	F = 1.53, *p* = 0.234, η^2^ = 0.24
Supraspinale (mm)	13 ± 5.6	7.1 ± 2.1	12.8 ± 6.4	13.7 ± 5.2	9.6 ± 6.2	F = 1.82, *p* = 0.167, η^2^ = 0.28
Subscapular (mm)	8.3 ± 2	9 ± 1.6	11.2 ± 3.8	11.5 ± 5.3	10.2 ± 1.6	F = 0.67, *p* = 0.622, η^2^ = 0.12
Calf (mm)	10.8 ± 1.5	12.7 ± 3.4	13 ± 2.5	14.1 ± 3.7	11.3 ± 1.3	F = 0.66, *p* = 0.628, η^2^ = 0.12
	Somatotypes					
Endomorphy	3.4 ± 0.3	2.9 ± 0.6	3.9 ± 1	3.6 ± 1.1	2.8 ± 1.1	F = 1.43, *p* = 0.264, η^2^ = 0.23
Mesomorphy	1.2 ± 1.9	3.4 ± 0.9	2.8 ± 0.8	2.9 ± 0.8	4.6 ± 0.5 ^a^	F = 3.78, ***p* = 0.020**, η^2^ = 0.44
Ectomorphy	4.1 ± 0 ^c^	2.7 ± 1	2 ± 0.7	2.5 ± 0.9	2.6 ± 1	F = 2.39, *p* = 0.087, η^2^ = 0.34

Legend: M—Mean; SD—standard deviation; different from: ^a^—<50; ^b^—<55; ^c^—<61; ^d^—<68; ^e^—>68; significant differences in bold.

## Data Availability

The data presented in this study are available on request from the corresponding author.
